# Can Dialysis Patients Identify and Diagnose Pulmonary Congestion Using Self-Lung Ultrasound?

**DOI:** 10.3390/jcm12113829

**Published:** 2023-06-02

**Authors:** Eyal Schneider, Netta Maimon, Ariel Hasidim, Alla Shnaider, Gabrielle Migliozzi, Yosef S. Haviv, Dor Halpern, Basel Abu Ganem, Lior Fuchs

**Affiliations:** 1 Faculty of Health Sciences, Ben-Gurion University of the Negev, Beer-Sheva 7747629, Israel; ariel.hasidim@gmail.com (A.H.); migliozz@post.bgu.ac.il (G.M.); halpern.dor1@gmail.com (D.H.); liorfuchs@gmail.com (L.F.); 2 Department of Nephrology, Soroka University Medical Center, Beer-Sheva 8457108, Israel; ellasc@bgu.ac.il (A.S.); havivy@bgu.ac.il (Y.S.H.); 3 Department of Health Policy and Management, Ben-Gurion University of the Negev, Beer-Sheva 7747629, Israel; 19basl@gmail.com; 4 Emergency Room, Joseftal Hospital, Eilat 8808024, Israel; 5 Medical Intensive Care Unit and Clinical Research Center, Soroka University Medical Center, Beer-Sheva 8457108, Israel

**Keywords:** point of care ultrasound (POCUS), lung ultrasound (LUS), self-ultrasound, dialysis, pulmonary congestion

## Abstract

Background: With the recent developments in automated tools, smaller and cheaper machines for lung ultrasound (LUS) are leading us toward the potential to conduct POCUS tele-guidance for the early detection of pulmonary congestion. This study aims to evaluate the feasibility and accuracy of a self-lung ultrasound study conducted by hemodialysis (HD) patients to detect pulmonary congestion, with and without artificial intelligence (AI)-based automatic tools. Methods: This prospective pilot study was conducted between November 2020 and September 2021. Nineteen chronic HD patients were enrolled in the Soroka University Medical Center (SUMC) Dialysis Clinic. First, we examined the patient’s ability to obtain a self-lung US. Then, we used interrater reliability (IRR) to compare the self-detection results reported by the patients to the observation of POCUS experts and an ultrasound (US) machine with an AI-based automatic B-line counting tool. All the videos were reviewed by a specialist blinded to the performer. We examined their agreement degree using the weighted Cohen’s kappa (Kw) index. Results: A total of 19 patients were included in our analysis. We found moderate to substantial agreement between the POCUS expert review and the automatic counting both when the patient performed the LUS (Kw = 0.49 [95% CI: 0.05–0.93]) and when the researcher performed it (Kw = 0.67 [95% CI: 0.67–0.67]). Patients were able to place the probe in the correct position and present a lung image well even weeks from the teaching session, but did not show good abilities in correctly saving or counting B-lines compared to an expert or an automatic counting tool. Conclusions: Our results suggest that LUS self-monitoring for pulmonary congestion can be a reliable option if the patient’s count is combined with an AI application for the B-line count. This study provides insight into the possibility of utilizing home US devices to detect pulmonary congestion, enabling patients to have a more active role in their health care.

## 1. Introduction

POCUS has evolved over the last few decades, transforming from a large, cumbersome machine with limited diagnostic capabilities to a modern lightweight device capable of providing a wealth of diagnostic information [1,2].

Initially, POCUS was limited to static visual inspection and anatomical measurements. Now, US machines offer displays in real time, enabling functional assessments [2,3] that further assist physicians in making quicker, more accurate diagnoses and in monitoring the effectiveness of treatments in real time [4,5].

The capabilities of POCUS are expanding to include a variety of automatic tools, including AI and a 5G network [1,3,6,7], improving our ability to conduct POCUS tele-guidance [8,9,10,11]. Telemedicine is proven to aid in managing patients with various chronic diseases [12,13]. Today, affordable US probes that work with cellular phones allow patients to monitor themselves remotely [14,15]. In addition, these innovative US devices are smaller and cheaper without compromising accuracy [16].

Cumulative evidence regarding the efficacy of LUS has established it as a reliable tool for detecting pulmonary congestion in HD patients [17,18,19,20], a common cause of hospital admission, cardiac events, and mortality [21]. Pulmonary congestion not only impairs respiratory function but also imposes an added burden on the cardiovascular system. It serves as a marker for volume overload, the most common complication of ESRD patients, contributing to the onset or progression of congestive heart failure, hypertension, and pulmonary hypertension [21,22]. Regular monitoring of extravascular lung water can aid in the early detection of pulmonary complications and guide treatment decisions. The LUST trial and its subsequent sub-trial have demonstrated that utilizing LUS to assess dry weight can improve clinical outcomes. These outcomes include lowered blood pressure, improved cardiac chamber dimensions, and enhanced left ventricular diastolic function [23,24,25,26]. A crucial advantage of this approach is that it avoids the adverse effects of depleting intravascular volume [27]. LUS is sensitive in detecting subclinical and asymptomatic pulmonary congestion [28,29,30,31,32], even days before pulmonary congestion clinically manifests [29]. Studies show that LUS is more accurate in detecting pulmonary congestion than relying on the presence of lung crackles and can detect even mild changes in extravascular lung water EVLW [26,33].

Pulmonary congestion is diagnosed on LUS by detecting ultrasonographic B-lines [28]. B-lines are vertical hyperechoic reverberation artifacts that appear on imaging. They result from the US beam’s reflection on subpleural, fluid-thickened interlobular septa [34]. Identifying B-lines using LUS is relatively simple to teach and conduct [31,35]. Moreover, B-lines can be counted for a B-line score (BLS) [30,31]. This method offers a more objective and quantifiable approach for evaluating EVLW and diagnosing pulmonary congestion.

The use of automated techniques for LUS imaging has developed dramatically in recent years, with a rapid increase in operation and understanding of such tools during the COVID-19 pandemic [36,37,38,39,40]. Automatic tools such as computer-aided diagnosis systems and image analysis algorithms have made it possible to quickly and accurately analyze images. Research has demonstrated the efficacy of several programmed models in evaluating B-lines and their correlation to various clinical conditions [41,42,43,44], including HD [45]. Software algorithms can detect and count B-lines automatically in real time, providing a reliable option for less experienced users [6,34,46].

Only two studies compared self-monitoring to professional monitoring in HF patients and healthy subjects. The researchers revealed a high expert agreement with patients’ scans, thus supporting the capability of patients to self-recognize B-lines and leaving room for further investigation [15,47].

There are many advantages to LUS bedside assessment: it is easy and rapid to perform, mobile, non-invasive, low-cost, and has a relatively short learning curve [28,29,35,48,49,50]. Studies show that achieving a reasonable degree of competency is possible after a brief training process [29,30,35]. In addition, by early self-identification of evolving pulmonary congestion, a tool such as LUS can assist patients in better management of their disease. It may shorten the time to definitive treatment [29,49], potentially reduce hospitalizations, and improve clinical outcomes.

In this present study, we sought to assess the self-detection of pathological B-lines by estimating both the scanning and B-line counting ability of HD patients, performing lung ultrasound on themselves, with and without an AI-based B-line tool. The results reported by the patients were compared to POCUS expert assessment and to the AI tool count ability. We hope that this research can provide a foundation for future studies that will investigate the potential of LUS in supporting the remote evaluation of patients.

## 2. Materials and Methods

### 2.1. Participants and Setting

This was a prospective pilot single-center study conducted at the nephrology ward at SUMC, Be’er Sheva, Israel, from November 2020 to September 2021.

The patients were recruited from the dialysis clinic using a convenient sampling method, as this study was conducted as a pilot study (Table 1). Subjects were included in the study if they received chronic dialysis, were physically capable of performing US on themselves, had a smartphone, used a messaging application (this criterion is designed to ensure that the patients participating in the study have basic technological capabilities), agreed to participate, were cognitively intact (this was judged by the treating nephrologist), and could sign an informed consent. Subjects were excluded from the study if they were morbidly obese (BMI > 35) or had a lung resection or lung transplant history.

**Figure 1:**
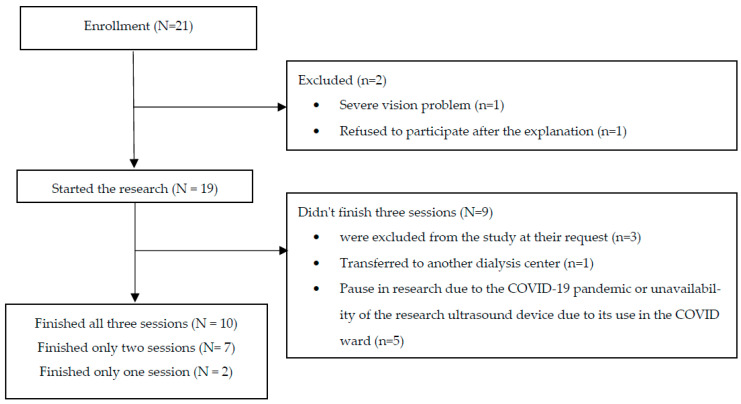
Flow chart for enrollment throughout the study.

Patients who fulfilled all the inclusion criteria and none of the exclusion criteria were approached by a study research member and offered to take part in the study (Figure 1). The purpose, procedures, and study objectives were explained to each potential participant. The patients were asked to sign an informed consent upon agreeing to participate in the study. Subsequently, they were taught to self-scan the lungs before and after dialysis treatment, as detailed below. The hospital’s ethical committee approved the study (0218-20-SOR).

### 2.2. Study Flow

US examinations were conducted using the Venue Go TM, a GE Healthcare US device with the cardiac 3Sc-RS probe (3 MHz), which is suitable for the lung scan and identification of B-lines when using the appropriate preset settings designated for lung scanning (FPS 31, F 3.6, P 0 dB, Dynamic range 60 dB, Depth 16 cm, and Gain 0 dB); this US device has auto-B-lines detection capabilities that were validated in a previous study [6]. The study plan involved meeting each patient for three dialysis treatments, during which a scan in each treatment would be performed before and after the dialysis. The patient would perform scans in two zones, while the researcher would perform in four zones. In the first session, participants were taught to use the US by holding the probe on the mid-clavicular line at zone 1. This zone was selected to simplify the procedure and because the anterior zones have high sensitivity and specificity for detecting B-lines [51,52]. They also received a brochure explaining B-lines and how to identify and count them using examples with images. The brochure was translated into all relevant languages and was provided to each patient to review at home and before scanning during each dialysis visit (Appendix A).

**Figure 2:**
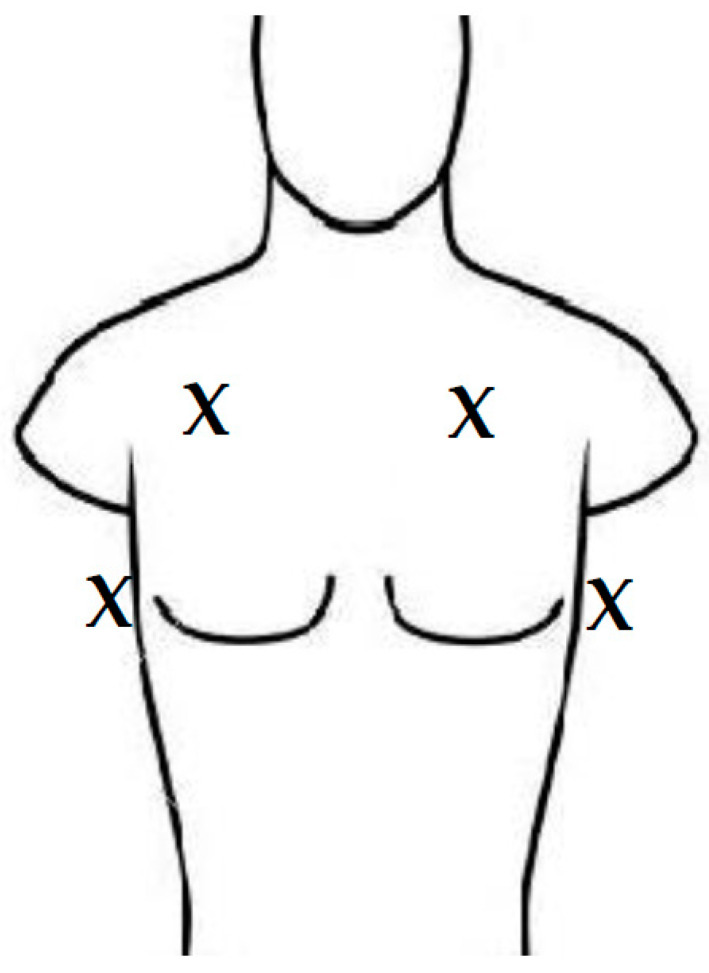
Researchers’ scan locations.

At the 1st visit, after the teaching session, the patients were asked to identify the number of B-lines on four separate printed images. This was conducted to ensure patients understood B-lines and how to identify them. The scan was performed on both anterior chest sides unless there was a subclavian dialysis catheter on one side; in this case, the scan was not performed on that side. In the following sessions, the participants performed the scan independently and counted the number of B-lines without any reminder or instruction from the study member. At each visit, the research team saved LUS clips from the participants’ clips once the patient was satisfied with the images. The researcher performed scans in the same locations as the patient and in the mid-axillary region on both sides (Figure 2). To avoid bias, the researchers’ scans were acquired only after the patients’ scans. Images from researchers’ clips were also recorded and stored on the US machine. Before the clips were saved, the number of B-lines from all scans was counted by the automatic tool and kept in the device memory.

Once all the scans were collected, an experienced physician (LF 10 years) operator, blind to the performer (researcher or patient), reviewed all the clips. The reviewer assessed and counted B-lines independently, blinded to the automatic tool count and patient count. Additionally, the reviewer estimated the performer’s ability to correctly capture the lung ultrasonographic anatomy: the identification of a clear, centered, pleural line surrounded by two acoustic shadows. This evaluation was conducted on a scale of 0 to 2 for both researchers and patients. The lung scans were recorded and ranked in the following categories: 0 (poor)—the anatomical capture is not clear enough for B-line detection, 1 (good)—the anatomical capture is not perfect, but the reviewer can assess B-lines with moderate confidence, and 2 (excellent)—the ideal anatomical markers of a narrow pleural line with two acoustic shadows on either side (rib space) can be visualized, and the reviewer can assess B-lines with confidence.

Data collection for this study included demographic factors such as age, gender, ethnicity, years of education, and native language, as well as medical history information, including medications and chronic diseases. Clinical data included dates of admission, treatment such as dialysis duration (years) and the number of treatments per week, vital signs, and dry weight. Finally, US results were collected, including BLS detected from scans collected by the patient from two zones, and four zones by the researchers. B-line counts were collected from four different sources: the patient, the researcher, the expert, and the AI automatic tool.

### 2.3. Statistical Analysis

Since our reported observation had three ordinal levels, we used weighted Cohen’s kappa to assess the observers’ agreement and weigh the disagreements differently. We report Kw statistics and 95% confidence intervals, and compare them in an inter-observer kappa agreement matrix. The expert’s count on clips captured by the researcher will be defined as the “gold standard”. Kw statistics will be interpreted as follows: Kw = 0 (good as a guess), Kw = 0.01–0.2 (slight agreement), Kw = 0.21–0.4 (fair agreement), Kw = 0.41–0.6 (moderate agreement), Kw = 0.61–0.80 (substantial agreement), Kw = 0.81–0.99 (near-perfect agreement), and Kw = 1.00 (perfect agreement).

The analysis was conducted using the R statistical programming language, version 4.1.2.

## 3. Results

### 3.1. General Information

Twenty-one patients were recruited for the study. Two were excluded due to severe vision problems and refused to participate (Figure 1). A total of 283 clips was reviewed, 137 that were saved by the patients and 146 that were saved by the researchers. Of the 19 patients enrolled in the study, 12 (63%) were men, and the average age was 65.7 years. Most patients received three dialysis treatments per week, and a few underwent four. Most patients are Jewish (17, 89%) and native Hebrew speakers (16, 84%). In total, 7 (37%) have diabetes, 16 (84%) have high blood pressure, 7 (37%) have ischemic heart disease, and 2 (10%) have previously undergone a kidney transplant (Table 1).

### 3.2. B-Line Count Agreement

There was a moderate agreement between the gold standard (expert count on researcher’s clip) and the patients’ auto-B-line count clip (N = 91, Kw = 0.49 [95% CI: 0.05–0.93]). There was a substantial agreement between the gold standard clip and the auto-B-line count from the researcher’s scan (N = 241, Kw = 0.67 [95% CI: 0.67–0.67]). There was an insignificant slight agreement between the patients’ count on self-clips and the gold standard (expert count on researcher’s clip) (N = 129, Kw = 0.2 [95% CI: −0.89–1.0]; Table 2. There was a fair agreement between the expert’s count on the patients’ clips and the gold standard, but without statistical significance (N = 69, Kw = 0.3 [95% CI: −0.76–1.0]) (Table 2).

### 3.3. Correct Lung Image

To test the patients’ ability to place the probe in the correct position and capture a reliable image with a good ultrasonographic anatomy view of the lung, the expert rated each patient’s video according to the described three categories. The quality results of the clips in the first session were as follows (Table 3): 12 (21.8%) poor, 12 (21.8%) good, and 31 (56.4%) excellent, with an average score of 1.35 (from a scale of 0 to 2). In the second session, 18 (38.3%) were poor, 16 (34%) were good, and 13 (27.7%) were excellent; the average score was 0.89. In the third session, 15 (42.9%) videos were poor, 8 (22.9%) were good, and 12 (34.3%) were excellent. The average score in the third session was 0.91. In all three sessions, the rates of good or excellent were achieved by more than 50% of the patients. This was achieved without training beyond the first session.

The lung scans were recorded and ranked in the following categories: 0 (poor)—the anatomical capture is not clear enough for B-line detection, 1 (good)—the anatomical capture is not perfect, but the reviewer can assess B-lines with moderate confidence, and 2 (excellent)—the ideal anatomical markers of a narrow pleural line with two acoustic shadows on either side (rib space) can be visualized, and the reviewer can assess B-lines with confidence.

### 3.4. Schedules

The mean time interval between enrollment and the second session was 16.44 days (SD 12.9). This is very similar to the interval between the second session and the third session, which was 16.4 days (SD 10.3). However, the maximum interval between enrollment and the second session was 40 days with a minimum of 2 days compared to a maximum interval of 32 days and a minimum of 3 days between the second and third sessions (Table 4).

## 4. Discussion

The primary objective of this study was to determine whether self-performed LUS for B-lines could produce reliable results useful for clinical evaluation. By comparing BLS counted by the patients to BLS counted by an expert on researcher clips (gold standard), our results indicate that with the assistance of a live automatic BLS counting tool, patients are able to report the number of B-lines with moderate accuracy (Kw = 0.49, Table 2). Regarding the quality of the scans, most patients achieved a sufficient lung anatomical ultrasonographic window, defined as a good or excellent anatomical capture, even weeks after their primary and only learning session. In the first session, the success rate for at least good self-lung US imaging was 78% for patients versus 95% for researchers; in the second session, 62% versus 94%; and in the third session, 58% versus 99%, respectively. This reduction in patients’ competency is understandable as no further training was conducted throughout the trial. We believe that extra training should be performed in future studies for the consolidation of self-lung ultrasound scan competency. A plausible solution is to integrate an instructional video to guide the patient in performing a self-lung US scan in real time, as shown to be successful in a study that used a portable self-US device in pregnancy [14]. Additionally, a short reminder practice at the beginning of the teaching process during the first few outpatient clinic follow-ups could improve patients’ scanning ability until the patient performs sufficient scans in consecutive visits.

Our study strengthens the new concept that scans for B-lines can be achieved by patients, and we believe they can be conducted even at home in the future [45]. Results indicate a fair, however insignificant, agreement of 0.3 between the expert count on patient-acquired clips and the expert count on researcher-acquired clips (gold standard, Table 2). As described in the Section 2, the calculation of BLS by the AI is made in real time, independent of the clip chosen by the patients to save. This explains the discrepancy of the moderate agreement (Kw = 0.49) between the gold standard and patients’ auto-B-line count and the only fair agreement (Kw = 0.3) between the gold standard and expert count on the patient’s scan. Although patients performed the test with reasonable quality, they needed to be more adept in identifying and saving the clip when the image most closely reflected the correct number of B-lines. Therefore, there was inconsistency between the quality of the acquired LUS clips by the patients (which was graded as at least moderate) and the number of B-lines that were counted from the patients’ images compared to the number that was counted from a following LUS clip conducted by the researcher. The automatic B-line tool can offer a solution by allowing more time for the LUS recording, recognizing, and counting B-lines more accurately in real time. Indeed, when the automatic tool was activated on patients’ scans, we found a moderate agreement, 0.49, between the AI BLS count activated on patients’ scans and the expert count on the researcher’s clips (gold standard). These results imply that the AI count is pivotal for implementing patient self-testing of LUS for the detection of pulmonary congestion. Future studies should give more attention to the recording technique itself. This can be achieved by planning clips with longer duration or capturing additional clips in the same area. Furthermore, relying on the AI B-line count can serve as a checkpoint for appropriate recording timing or as an alternative for a post-hoc interpretation of recorded clips by the expert.

Our results demonstrate a slight and insignificant agreement, 0.08, between the patient and expert B-line count on patients’ scans. This suggests suboptimal counting ability by patients. At the same time, there is much better agreement between AI and the expert count on clips recorded by the patient and researcher, respectively, supporting the reliability of an AI-based B-line count. A recent study from our group validated the accuracy of the automatic B-line count tool used in this study [6]. This tool provides a more straightforward process for patients and clinicians. Patients can focus exclusively on obtaining an appropriate position for scanning without the effort of saving an image at the correct time or focusing on counting B-lines. AI can count the most identified B-lines tracked in a clip in real time and send the results automatically to a health care professional.

Incorporating practical usage of cellular-based, cheap US devices has several beneficial implications with the potential to guide earlier treatment, prevent exacerbations, and reduce emergency room visits and hospital admissions [12]. Our study aligns with the results of previous experimental studies that have investigated self-conducted US scans. For example, in two recent surveys, CHF patients and healthy participants were trained to perform a lung US self-exam successfully, with adequate interpretability of scans and high patient-reported self-efficacy and competence [15,47]. Additionally, a study using a portable self-US device in pregnancy concluded that it is an efficient, practical method for remote sonographic fetal assessment [12]. This progress has significant implications for enhancing patient care, especially considering the challenge of treating patients at home for evolving lung illness post-admission throughout the COVID-19 pandemic. LUS played a substantial role in the assessment of such patients in the community [53,54] in the acute setting [55] and the post-illness follow-up [56].

Several studies have shown the benefits of involving patients in their screening and follow-up process or transferring some responsibility for self-follow-up to patients [57]. For example, a review that combined conclusions from several studies on patients with diabetes found that those more actively involved in managing their condition had better blood sugar control and a lower risk of complications [58]. Another review of pediatric asthma found that those more involved in their care had better symptom control and a lower risk of hospitalization [59]. Additionally, involving patients in their care can lead to greater patient satisfaction and empowerment, as patients feel more in control of their health. This is especially important for patients with chronic conditions, who may be dealing with their disease over an extended period.

It should be emphasized that no correlation was observed between participants’ years of education or profession and the degree of success in performing LUS or counting the BLS. Therefore, studying the scan is suitable for a wide range of patients, and further inclusion or exclusion criteria are not required. The training of how to operate such modalities can extend to family members or hired caregivers.

There is potential for using cheap, portable devices connected to cell phones with automatic tools to improve the self-management of patients with chronic heart failure (CHF) and patients on dialysis. A meta-analysis in which remote monitoring of heart failure patients was assessed showed that it enabled faster intervention and was associated with lower mortality [60]. By conducting continuous, early home monitoring, patients might be able to detect life-threatening conditions such as PC earlier, potentially reducing the number of hospitalizations and improving outcomes. These devices could be handy for patients who live in rural or underserved areas where access to health care may be limited [61].

This research has several limitations. First, the recruitment ability was limited as a single-centered, non-sponsored, prospective study. Accordingly, there was an objective difficulty in recruiting a large number of patients, and therefore our sample size is small. As a result, in some statistical analyses, we encountered difficulty obtaining significant results and identifying factors that influence success or failure in learning to identify B-lines. Therefore, the statistical power is restricted as the external validity, and the hypothesis requires further investigation in future, more extensive studies.

The study sample only reflects part of the dialysis patient population. Because most of the patients in the region are treated in community dialysis institutes, many of the study participants treated in the medical center’s dialysis unit are relatively older and more medically complex. On the other hand, treatment in the hospital’s nephrology center is probably more extensive, so patients are better balanced and less likely to exhibit B-lines on scans. The generalizability of our findings is also limited because only patients using a messaging app on their smartphones were recruited.

Some of the recordings achieved by the patients mistakenly had only still pictures acquired instead of a video clip. This technical error limited our ability to assess image quality, count the number of B-lines, and perform the sub-analysis, thus reducing our sample size and impacting the significance of our findings. We believe that a designated application in a private cellular phone that automatically starts the scan when the US cradle is placed over the chest, with a designated automatic tool for the real-time B-line count, can overcome this limitation of correct clip capture.

Finally, there were long pauses in the recruitment phase of the study due to the COVID-19 pandemic, which might have influenced patients’ memory and performance, although the median time was 15.5 days between the first and second encounters and 13 days between the second and third encounters.

Our study has strengths. Only one other published study investigates patients’ ability to perform a self-LUS scan [47]. Our study is the first to measure patient performance in a self-LUS scan beyond one encounter and in a long significant period of time. Finally, it is the first study to combine and validate an AI-based automatic B-line count tool with patient self-lung scans for B-lines, with the demonstration of such tool advantage.

Involving patients in self-assessment using an imaging tool operated at home is a novel concept that enables patients to take on a more active and engaging role in their health care. Our findings are the substrate for future pilot studies where patients can receive cellular-based US machines delivered to their homes to test self-US lung scans away from the research team. This may progress POCUS from being solely performed by physicians in clinical and research settings to patients using US at home, participating in their clinical assessment and care [14].

## 5. Conclusions

Dialysis patients can self-scan their lungs with good anatomical precision, and when using an AI-based counting application, accurate B-line counts are comparable to an expert’s count on clips taken by researchers. We believe this study sheds light on the potential for using home US devices to detect PC, allowing patients to take a more active role in their medical care.

## Figures and Tables

**Table 1:** Baseline patient characteristics and demographic profile.

	Total (N = 19)
Gender	
Female	7 (36.8%)
Male	12 (63.2%)
Age, Mean (SD)	65.7 (14.3)
Years of education, Median [Min, Max]	12.0 [6.00, 20.0]
Missing	4 (21.1%)
Weekly dialysis treatments, Median [Min, Max]	3.00 [3.00, 4.00]
Native language	
Amharic	1 (5.3%)
Arabic	2 (10.5%)
Hebrew	16 (84.2%)
Religion	
Jewish	17 (89.5%)
Muslim	2 (10.5%)
First wet weight, Mean (SD)	77.3 (15.3)
DM	7 (36.8%)
Missing	2 (10.5%)
HTN	16 (84.2%)
Missing	2 (10.5%)
IHD	7 (36.8
Missing	2 (10.5%)
Kidney transplantation	2 (10.5%)

**Table 2:** B-line score comparison agreement.

	Patients’ Count	Expert’s Count on Patients’ Clip	Expert’s Count on Researcher’s Clip	Patients’ Auto-B-Line Count	Researcher’s Auto-B-Line Count
**Patients’ count**	1				
**Expert’s count, patient’s clip**	**0.08** [−1.0–1.0] (N = 58)	1			
**Expert’s count, researcher’s clip**	**0.20** [−0.89–1.0] (N = 129)	**0.30** [−0.76–1.0] (N = 69)	1		
**Patients’ auto-B-line count**	**0.22** [−0.75–1.0] (N = 71)	**0.49** [−0.16–1.0] (N = 74)	0.49 * [0.05–0.93] (N = 91)	1	
**Researcher’s auto-B-line count**	**0.07** [−1.0–1.0] (N = 110)	**0.26** [−0.74–1.0] (N = 69)	0.67 * [0.67–0.67] (N = 241)	**0.46** [−0.09–1.0] (N = 100)	1

Weighted kappa values (Kw) were used for the estimation of agreement between observers. In the study, clips were saved by the patients (patients’ clip) and by the researchers (researcher’s clip). All the clips of the patients and of the researchers were counted for B-lines by an expert doctor (expert’s count) and by the auto tool (auto-B-line count). The expert’s count on researchers’ clips was considered the “gold standard.” Please note that the calculation of BLS by the AI is made in real time, independent of the clip chosen by the patients to save. The * marks the results that were statistically significant with *p*-value < 0.05

**Table 3:** Lung image quality rank score comparison.

	Enrollment		Second Visit		Third Visit	
	Patient (N = 55)	Researcher (N = 62)	Patient (N = 47)	Researcher (N = 49)	Patient (N = 35)	Researcher (N = 35)
**Quality category**						
Poor	12 (21.8%)	3 (4.8%)	18 (38.3%)	3 (6.1%)	15 (42.9%)	0 (0%)
Good	12 (21.8%)	10 (16.1%)	16 (34.0%)	10 (20.4%)	8 (22.9%)	6 (17.1%)
Excellent	31 (56.4%)	49 (79.0%)	13 (27.7%)	36 (73.5%)	12 (34.3%)	29 (82.9%)
**Descriptive parameters**						
Mean (SD)	1.35 (0.821)	1.74 (0.541)	0.894 (0.814)	1.67 (0.591)	0.914 (0.887)	1.83 (0.382)
Median [Q1, Q3]	2.00 [1.00, 2.00]	2.00 [2.00, 2.00]	1.00 [0, 2.00]	2.00 [1.00, 2.00]	1.00 [0, 2.00]	2.00 [2.00, 2.00]

**Table 4:** Descriptive statistics for the time between enrollment and second visit, and second and third visits (in days).

EVENT_NAME	N	Mean	SD	25th	50th	75th	Min	Max
Enrollment and 2nd visit	16	16.4	12.9	4.25	15.5	28	2	40
2nd and 3rd visits	10	16.4	10.3	9.75	13.0	26	3	32

**Figure A1:**
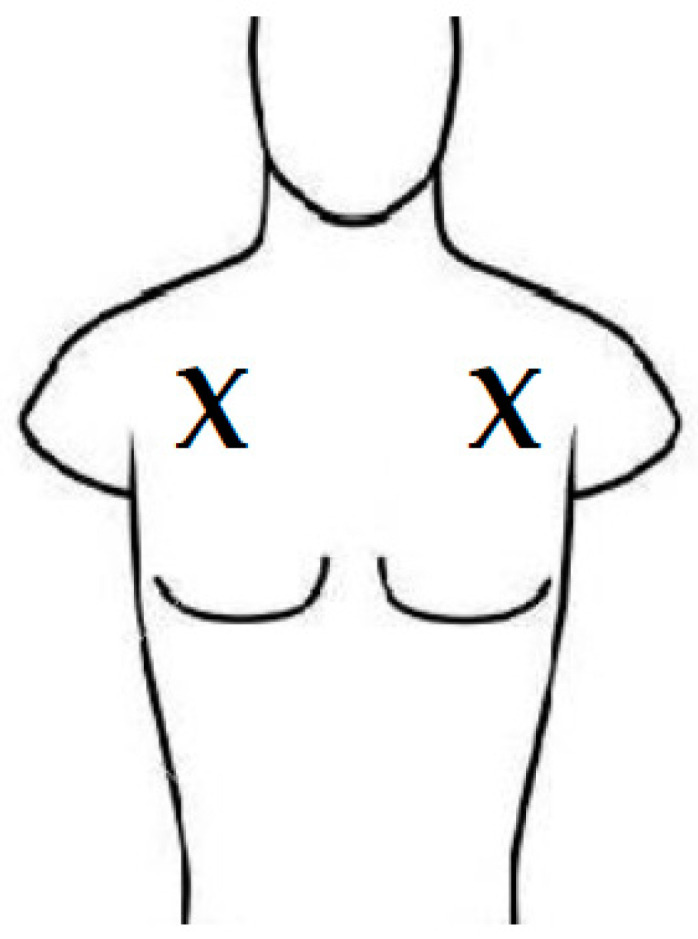
Patients’ scan locations, as illustrated in the explanatory brochure they received.

**Figure A2:**
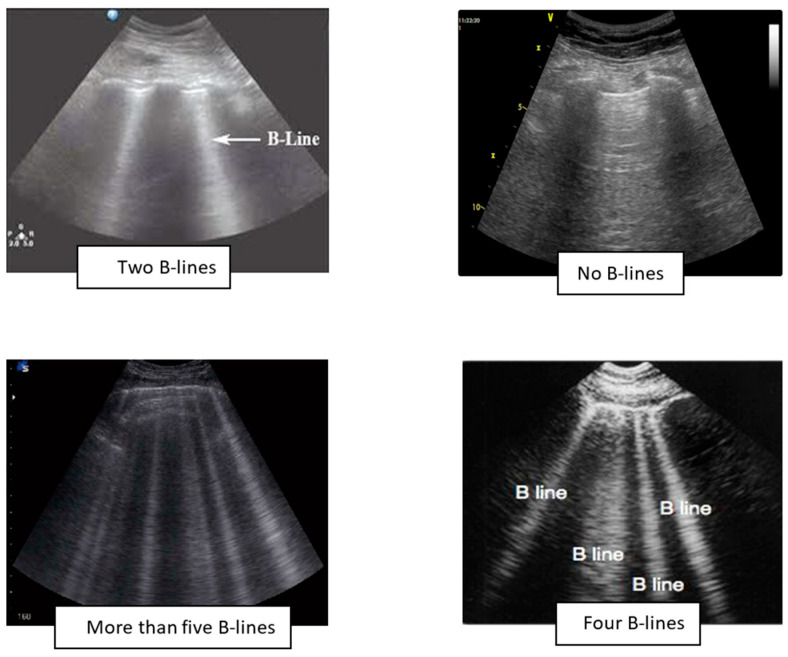
Examples of possible B-line images and appropriate interpretations, as illustrated in the patients’ explanatory brochure.

## Data Availability

The data presented in this study are available on request from the corresponding author.

## Appendix A

Ultrasound examination creates an image of the body’s organs using sound waves. The test is safe, does not cause damage, and therefore can be performed without limitation.

The test is performed using a transmitter–receiver called a transducer. The ultrasound device processes the sound waves received by the transducer into a live image displayed on the screen in real time. Since the presence of air interferes with the passage of sound waves, a gel is applied to the transducer, thus creating “direct contact” between the transducer and the skin.

**The test**: During the test, you will be asked to take a short video of the lungs.

You will hold the transducer in one hand; after a little gel has been applied to it, you will be asked to place it on one of the points marked in the figure. You can move the transducer slightly up or down until you obtain an image where you can see the space between the ribs and the lungs behind them.

Upon obtaining the image, you will be asked to count the number of B-lines in the image; an explanation is below.

After that, we will perform the test on the other side of the chest as well. In addition, we will repeat the test once more after the dialysis treatment.

**B-lines**: White or light gray lines starting at the top of the screen and reaching the bottom. They look similar to a curtain fluttering in the wind and can move with the movement of the device or during breathing. The B-lines can indicate the amount of fluid in the lungs.

Examples of possible images:

## References

[1] Barjaktarevic I., Kenny J.S., Berlin D., Cannesson M. (2021). The Evolution of Ultrasound in Critical Care: From Procedural Guidance to Hemodynamic Monitor. J. Ultrasound Med..

[2] Díaz-Gómez J.L., Mayo P.H., Koenig S.J. (2021). Point-of-Care Ultrasonography. N. Engl. J. Med..

[3] Yaoting W.M., Huihui C.M., Ruizhong Y.M., Jingzhi L.M.P., Ji-Bin L.M., Chen L., Chengzhong P. (2021). Point-of-Care Ultrasound: New Concepts and Future Trends. Adv. Ultrasound Diagn. Ther..

[4] Arnold M.J., Jonas C.E., Carter R.E. (2020). Point-of-Care Ultrasonography. Am. Fam. Physician.

[5] Ben-Baruch Golan Y., Sadeh R., Mizrakli Y., Shafat T., Sagy I., Slutsky T., Kobal S.L., Novack V., Fuchs L. (2020). Early Point-of-Care Ultrasound Assessment for Medical Patients Reduces Time to Appropriate Treatment: A Pilot Randomized Controlled Trial. Ultrasound Med. Biol..

[6] Tsaban G., Galante O., Almog Y., Ullman Y., Fuchs L. (2021). Feasibility of machine integrated point of care lung ultrasound automatic B-lines tool in the Corona-virus 2019 critical care unit. Crit. Care.

[7] Gohar E., Herling A., Mazuz M., Tsaban G., Gat T., Kobal S., Fuchs L. (2023). Artificial Intelligence (AI) versus POCUS Expert: A Validation Study of Three Automatic AI-Based, Real-Time, Hemodynamic Echocardiographic Assessment Tools. J. Clin. Med..

[8] Baribeau Y., Sharkey A., Chaudhary O., Krumm S., Fatima H., Mahmood F., Matyal R. (2020). Handheld Point-of-Care Ultrasound Probes: The New Generation of POCUS. J. Cardiothorac. Vasc. Anesth..

[9] Haji-Hassan M., Lenghel L.M., Bolboacă S.D. (2021). Hand-Held Ultrasound of the Lung: A Systematic Review. Diagnostics.

[10] Filopei J., Siedenburg H., Rattner P., Fukaya E., Kory P. (2014). Impact of pocket ultrasound use by internal medicine housestaff in the diagnosis of Dyspnea. J. Hosp. Med..

[11] Gustafsson M., Alehagen U., Johansson P. (2015). Imaging Congestion with a Pocket Ultrasound Device: Prognostic Implications in Patients with Chronic Heart Failure. J. Card. Fail..

[12] Ma Y., Zhao C., Zhao Y., Lu J., Jiang H., Cao Y., Xu Y. (2022). Telemedicine application in patients with chronic disease: A systematic review and meta-analysis. BMC Med. Inform. Decis. Mak..

[13] Timpel P., Oswald S., Schwarz P.E.H., Harst L. (2020). Mapping the Evidence on the Effectiveness of Telemedicine Interventions in Diabetes, Dyslipidemia, and Hypertension: An Umbrella Review of Systematic Reviews and Meta-Analyses. J. Med. Internet Res..

[14] Hadar E., Wolff L., Tenenbaum-Gavish K., Eisner M., Shmueli A., Barbash-Hazan S., Bergel R., Shmuel E., Houri O., Dollinger S. (2022). Mobile Self-Operated Home Ultrasound System for Remote Fetal Assessment During Pregnancy. Telemed. e-Health.

[15] Duggan N.M., Jowkar N., Ma I.W.Y., Schulwolf S., Selame L.A., Fischetti C.E., Kapur T., Goldsmith A.J. (2022). Novice-performed point-of-care ultrasound for home-based imaging. Sci. Rep..

[16] Prinz C., Voigt J.-U. (2011). Diagnostic Accuracy of a Hand-Held Ultrasound Scanner in Routine Patients Referred for Echocardiography. J. Am. Soc. Echocardiogr..

[17] Giannese D., Puntoni A., Cupisti A., Morganti R., Varricchio E., D’alessandro C., Mannucci C., Serio P., Egidi M.F. (2021). Lung ultrasound and BNP to detect hidden pulmonary congestion in euvolemic hemodialysis patients: A single centre experience. BMC Nephrol..

[18] Torino C., Tripepi R., Loutradis C., Sarafidis P., Tripepi G., Mallamaci F., Zoccali C. (2021). Can the assessment of ultrasound lung water in haemodialysis patients be simplified?. Nephrol. Dial. Transpl..

[19] Reisinger N., Lohani S., Hagemeier J., Panebianco N., Baston C. (2022). Lung Ultrasound to Diagnose Pulmonary Congestion Among Patients on Hemodialysis: Comparison of Full Versus Abbreviated Scanning Protocols. Am. J. Kidney Dis..

[20] Koratala A., Ronco C., Kazory A. (2020). The Promising Role of Lung Ultrasound in Assessment of Volume Status for Patients Receiving Maintenance Renal Replacement Therapy. Blood Purif..

[21] Zoccali C., Torino C., Tripepi R., Tripepi G., D’arrigo G., Postorino M., Gargani L., Sicari R., Picano E., Mallamaci F. (2013). Pulmonary Congestion Predicts Cardiac Events and Mortality in ESRD. J. Am. Soc. Nephrol..

[22] Loutradis C., Sarafidis P.A., Ferro C.J., Zoccali C. (2021). Volume overload in hemodialysis: Diagnosis, cardiovascular consequences, and management. Nephrol. Dial. Transplant..

[23] Loutradis C., Sarafidis P.A., Ekart R., Papadopoulos C., Sachpekidis V., Alexandrou M.E., Papadopoulou D., Efstratiadis G., Papagianni A., London G. (2019). The effect of dry-weight reduction guided by lung ultrasound on ambulatory blood pressure in hemodialysis patients: A randomized controlled trial. Kidney Int..

[24] Zoccali C., Torino C., Mallamaci F., Sarafidis P., Papagianni A., Ekart R., Hojs R., Klinger M., Letachowicz K., Fliser D. (2021). A randomized multicenter trial on a lung ultrasound–guided treatment strategy in patients on chronic hemodialysis with high cardiovascular risk. Kidney Int..

[25] Loutradis C., Papagianni A., Ekart R., Theodorakopoulou M., Minopoulou I., Pagourelias E., Douma S., Karagiannis A., Mallamaci F., Zoccali C. (2020). Excess volume removal following lung ultrasound evaluation decreases central blood pressure and pulse wave velocity in hemodialysis patients: A LUST sub-study. J. Nephrol..

[26] Torino C., Gargani L., Sicari R., Letachowicz K., Ekart R., Fliser D., Covic A., Siamopoulos K., Stavroulopoulos A., Massy Z.A. (2016). The Agreement between Auscultation and Lung Ultrasound in Hemodialysis Patients: The LUST Study. Clin. J. Am. Soc. Nephrol..

[27] Alexandrou M.-E., Theodorakopoulou M.P., Sarafidis P.A. (2022). Lung Ultrasound as a Tool to Evaluate Fluid Accumulation in Dialysis Patients. Kidney Blood Press. Res..

[28] Mallamaci F., Benedetto F.A., Tripepi R., Rastelli S., Castellino P., Tripepi G., Picano E., Zoccali C. (2010). Detection of Pulmonary Congestion by Chest Ultrasound in Dialysis Patients. JACC Cardiovasc. Imaging.

[29] Saad M.M., Kamal J., Moussaly E., Karam B., Mansour W., Gobran E., Abbasi S.H., Mahgoub A., Singh P., Hardy R. (2018). Relevance of B-Lines on Lung Ultrasound in Volume Overload and Pulmonary Congestion: Clinical Correlations and Outcomes in Patients on Hemodialysis. Cardiorenal Med..

[30] Ross D.W., Abbasi M.M., Jhaveri K.D., Sachdeva M., Miller I., Barnett R., Narasimhan M., Mayo P., Merzkani M., Mathew A.T. (2018). Lung ultrasonography in end-stage renal disease: Moving from evidence to practice-a narrative review. Clin. Kidney J..

[31] Martindale J.L. (2016). Resolution of sonographic B-lines as a measure of pulmonary decongestion in acute heart failure. Am. J. Emerg. Med..

[32] Miglioranza M.H., Picano E., Badano L.P., Sant’Anna R., Rover M., Zaffaroni F., Sicari R., Kalil R.K., Leiria T.L., Gargani L. (2017). Pulmonary congestion evaluated by lung ultrasound predicts decompensation in heart failure outpatients. Int. J. Cardiol..

[33] Vitturi N., Dugo M., Soattin M., Simoni F., Maresca L., Zagatti R., Maresca M.C. (2014). Lung ultrasound during hemodialysis: The role in the as-sessment of volume status. Int. Urol. Nephrol..

[34] Pičuljan A., Šustić M., Brumini G., Kuharić J., Šustić A. (2020). Reliability of B-line quantification by different-level observers and a software algorithm using point-of-care lung ultrasound. J. Clin. Monit. Comput..

[35] Gargani L., Sicari R., Raciti M., Serasini L., Passera M., Torino C., Letachowicz K., Ekart R., Fliser D., Covic A. (2016). Efficacy of a remote web-based lung ultrasound training for nephrologists and cardiologists: A LUST trial sub-project. Nephrol. Dial. Transplant..

[36] Guarracino F., Vetrugno L., Forfori F., Corradi F., Orso D., Bertini P., Ortalda A., Federici N., Copetti R., Bove T. (2021). Lung, Heart, Vascular, and Diaphragm Ultrasound Examination of COVID-19 Patients: A Comprehensive Approach. J. Cardiothorac. Vasc. Anesth..

[37] Mohamed Ali A.M., El-Alali E., Weltz A.S., Rehrig S.T. (2021). Thoracic Point-of-Care Ultrasound: A SARS-CoV-2 Data Repository for Future Artificial Intelligence and Machine Learning. Surg. Innov..

[38] Gutsche H., Lesser T.G., Wolfram F., Doenst T. (2021). Significance of Lung Ultrasound in Patients with Suspected COVID-19 Infection at Hospital Admission. Diagnostics.

[39] Brenner D.S., Liu G.Y., Omron R., Tang O., Garibaldi B.T., Fong T.C. (2021). Diagnostic accuracy of lung ultrasound for SARS-CoV-2: A retrospective cohort study. Ultrasound J..

[40] Demi L., Wolfram F., Klersy C., De Silvestri A., Ferretti V.V., Muller M., Miller D., Feletti F., Wełnicki M., Buda N. (2022). New International Guidelines and Consensus on the Use of Lung Ultrasound. J. Ultrasound Med..

[41] Moshavegh R., Hansen K.L., Moller-Sorensen H., Nielsen M.B., Jensen J.A. (2019). Automatic Detection of B-Lines in In Vivo Lung Ultra-sound. IEEE Trans. Ultrason. Ferroelectr. Freq. Control.

[42] Wang Y., Zhang Y., He Q., Liao H., Luo J. (2022). Quantitative Analysis of Pleural Line and B-Lines in Lung Ultrasound Images for Severity Assessment of COVID-19 Pneumonia. IEEE Trans. Ultrason. Ferroelectr. Freq. Control.

[43] Hu Z., Liu Z., Dong Y., Liu J., Bin Huang B., Liu A., Huang J., Pu X., Shi X., Yu J. (2021). Evaluation of lung involvement in COVID-19 pneumonia based on ultrasound images. Biomed. Eng. Online.

[44] Brusasco C., Santori G., Bruzzo E., Trò R., Robba C., Tavazzi G., Guarracino F., Forfori F., Boccacci P., Corradi F. (2019). Quantitative lung ultrasonography: A putative new algorithm for automatic detection and quantification of B-lines. Crit. Care.

[45] Tan G.F.L., Du T., Liu J.S., Chai C.C., Nyein C.M., Liu A.Y.L. (2022). Automated lung ultrasound image assessment using artificial intelligence to identify fluid overload in dialysis patients. BMC Nephrol..

[46] Short J., Acebes C., Rodriguez-de-Lema G., La Paglia G.M.C., Pavón M., Sánchez-Pernaute O., Vazquez J.C., Romero-Bueno F., Garrido J., Esperanza Naredo E. (2019). Visual versus automatic ultra-sound scoring of lung B-lines: Reliability and consistency between systems. Med. Ultrason..

[47] Chiem A.T., Lim G.W., Tabibnia A.P., Takemoto A.S., Weingrow D.M., Shibata J.E. (2021). Feasibility of patient-performed lung ultrasound self-exams (Patient-PLUS) as a potential approach to telemedicine in heart failure. ESC Heart Fail..

[48] Liang X.K., Li L.J., Wang X.H., Wang X.X., Wang Y.D., Xu Z.F. (2019). Role of Lung Ultrasound in Adjusting Ultrafiltration Volume in He-modialysis Patients. Ultrasound Med. Biol..

[49] Miglioranza M.H., Gargani L., Sant’Anna R.T., Rover M.M., Martins V.M., Mantovani A., Weber C., Moraes M.A., Feldman C.J., Kalil R.A.K. (2013). Lung Ultrasound for the Evaluation of Pulmonary Congestion in Outpatients. JACC Cardiovasc. Imaging.

[50] Balasubramaniam S., Annamalai I., Fernando M.E., Srinivasaprasad N.D., Suren S., Thirumalvalavan K., Veerakumar A.M. (2019). Volume assessment in hemodialysis: A comparison of present methods in clinical practice with sonographic lung comets. Indian J. Nephrol..

[51] Roshandel J., Alahyari S., Khazaei M., Asgari R., Moharamzad Y., Zarei E., Taheri M.S. (2021). Diagnostic performance of lung ultrasound com-pared to CT scan in the diagnosis of pulmonary lesions of COVID-19 induced pneumonia: A preliminary study. Virusdisease.

[52] Fuchs L., Galante O., Almog Y., Dayan R.R., Smoliakov A., Ullman Y., Shamia D., Ohayon R.B.D., Golbets E., El Haj K. (2022). Point of Care Lung Ultrasound Injury Score—A simple and reliable assessment tool in COVID-19 patients (PLIS I): A retrospective study. PLoS ONE.

[53] Bonnel A.R., Baston C.M., Wallace P., Panebianco N., Kinosian B. (2019). Using Point-of-Care Ultrasound on Home Visits: The Home-Oriented Ultrasound Examination (HOUSE). J. Am. Geriatr. Soc..

[54] Nouvenne A., Ticinesi A., Parise A., Prati B., Esposito M., Cocchi V., Crisafulli E., Volpi A., Rossi S., Bignami E.G. (2020). Point-of-Care Chest Ultrasonography as a Diagnostic Resource for COVID-19 Outbreak in Nursing Homes. J. Am. Med. Dir. Assoc..

[55] Barnikel M., Alig A.H.S., Anton S., Arenz L., Bendz H., Fraccaroli A., Götschke J., Vornhülz M., Plohmann P., Weiglein T. (2022). Follow-up lung ultrasound to monitor lung failure in COVID-19 ICU patients. PLoS ONE.

[56] Gurbani N., Acosta-Sorensen M., Díaz-Pérez D., Figueira-Goncalves J.M., Ramallo-Fariña Y., Trujillo-Castilla J.L. (2022). Clinical outcomes and lung ultrasound findings in COVID-19 follow up: Calm comes after the storm?. Respir. Med. Res..

[57] Allegrante J.P., Wells M.T., Peterson J.C. (2019). Interventions to Support Behavioral Self-Management of Chronic Diseases. Annu. Rev. Public Health.

[58] Lambrinou E., Hansen T.B., Beulens J.W. (2019). Lifestyle factors, self-management and patient empowerment in diabetes care. Eur. J. Prev. Cardiol..

[59] Harris K.M., Kneale D., Lasserson T.J., McDonald V.M., Grigg J., Thomas J. (2015). School-based self management interventions for asthma in children and adolescents: A mixed methods systematic review. Cochrane Database Syst. Rev..

[60] Nakamura N., Koga T., Iseki H. (2014). A meta-analysis of remote patient monitoring for chronic heart failure patients. J. Telemed. Telecare.

[61] Shaddock L., Smith T. (2022). Potential for Use of Portable Ultrasound Devices in Rural and Remote Settings in Australia and Other Developed Countries: A Systematic Review. J. Multidiscip. Health.

